# The rare presentations of a large polyp and an esophageal carcinoma in heterotropic gastric mucosa: a case series

**DOI:** 10.1186/1752-1947-1-127

**Published:** 2007-11-02

**Authors:** Hakan Alagozlu, Meltem Ergun, Mehmet Cindoruk, Selahattin Unal, Sukru Dumlu, Aylar Poyraz, Ayse Dursun

**Affiliations:** 1 Department of Internal Medicine, Faculty of Medicine, Division of Gastroenterology, Gazi University Hospital, Ankara, Turkey; 2 Department of Pathology, Faculty of Medicine, Gazi University Hospital, Ankara, Turkey

## Abstract

**Background:**

Heterotopic gastric mucosa (HGM) is commonly seen in the upper esophagus during endoscopyand is generally considered a benign disease. A hyperplastic polyp and an adenocarcinoma arising in heterotopic gastric mucosa are quite rare occurences.

**Case presentations:**

We present two cases: The first is a patient who suffered from dysphagia because of a large hyperplastic polyp that arose from HGM; the polyp was excised endoscopically. Secondly, we report a rare case of adenocarcinoma arising in HGM of the cervical esophagus.

**Conclusion:**

Morphologic changes or malignant transformation can develop in the inlet patch. Therefore, gastroenterologists should be aware of the possibility of HGM just distal to the upper esophageal sphincter.

## Background

Heterotopic gastric mucosa (HGM) in the cervical esophagus appears to result from incomplete replacement of the original columnar epithelium by stratified squamous epithelium in the embryonal period. HGM is found throughout the gastrointestinal tract, the most common site being the cervical esophagus. In endoscopic examination, HGM is frequently seen as a patchy lesion that is salmon or red colored with a sharp border. Macroscopically visible islands of HGM, referred as "inlet patches" are often detected during endoscopic examination. There are morphologic changes (benign complications such as stricture, ulcer, polyp, web, stenosis, fistula) in patients diagnosed with HGM III according to the clinicopathologic classification of esophageal HGM. Malignant transformation via dysplasia and intraepithelial neoplasia (HGM IV) and cervical esophageal adenocarcinoma of the HGM (HGM V) are exceedingly rare as are polyps in the HGM [1,2].

We present the case of a patient who suffered from dysphagia due to a large hyperplastic polyp that arose from HGM; the polyp was excised endoscopically. Also, we report a rare case of a patient with an adenocarcinoma arising in HGM of the cervical esophagus.

## Case presentations

### Case 1

A 55-year-old man presented with intermittent dysphagia of two months duration. His dysphagia especially involved solid foods and appeared localized in the upper esophagus. He didn't have any other gastrointestinal disorders including reflux disease. He also suffered from chronic renal failure and hepatitis C. He had been attending a hemodialysis program thrice weekly, but treatment for hepatitis C had not yet been started. He didn't smoke tobacco or drink alcohol.

**Figure 1:**
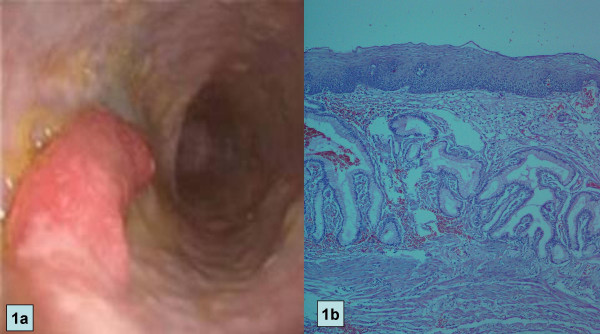
a. Endoscopic view of esophageal polyp which is located about 19 cm from the incisor teeth. b. Photomicrograph of polypectomy material, showing gastric mucosa with foveolar hyperplasia and intestinal metaplasia

An upper gastrointestinal endoscopy revealed a 5 cm polyp located 19 cm from the incisor teeth (Fig. 1a). Although the polyp was thought to be benign, in light of the symptoms of dysphagia, polypectomy was performed.

Polypectomy was performed on an inpatient basis, due to the concomitant diseases. The patient underwent hemodialysis without heparin. The procedure was done with topical anesthetic and intravenous meperidine (30 mg). An upper gastrointestinal endoscopy was performed with a standard forward-viewing videoendoscope (GIF-Q145, Olympus Optical Co. Ltd. Tokyo, Japan). After submucosal injection of diluted epinephrine (1:100 000), a snare polypectomy was performed using a monofilament polypectomy snare. The resection was performed using the ERBE-ICC 200 cautery device (ERBE Elektromedizin Gmgh, Tubingen, Germany). No residual polyp was visible at the polypectomy site. There were no complications.

The resection specimen was a sessile polyp (5 × 1 × 1 cm), with a granulated surface. Microscopically, it was a hyperplastic polyp consisting of gastric mucosa and intestinal metaplasia. There was no evidence of malignancy (Fig. 1b).

### Case 2

**Figure 2:**
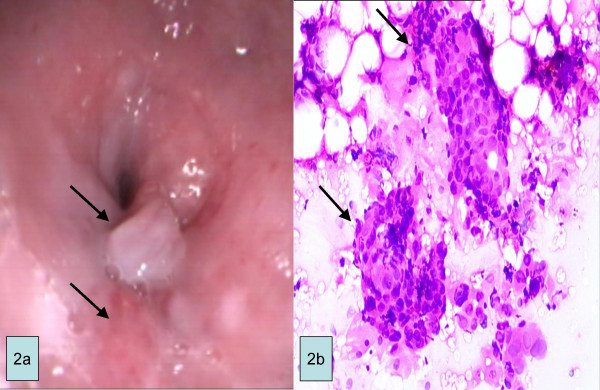
a. Endoscopic view of esophageal polypoid mass adjacent to reddish-colored epithelium (HGM). b. Photomicrograph showing malign epithelial cell islands in an inlet patch. (H&E, orig. mag. ×40)

A 57-year-old man underwent upper endoscopy because of odynophagia, dysphagia, nausea and vomiting. His past medical history was unremarkable. The patient did not report any symptoms suggestive of reflux disease in the preceding years. He had no relevant past or family history. His laboratory tests were normal except his hemoglobin (11,3 mg/dl). The endoscopic examination revealed a circular area of reddish-appearing mucosa from 21 to 22 cm in the esophagus with polypoid sessile bulgy lesions (each 2–3 cm in diameter) at the anterior wall (Fig. 2a). Endoscopy disclosed a submucosal bulgy tumor, covered with almost normal mucosa. Therefore, fine needle aspiration (FNA) was performed with endoscopic ultrasonography (EUS). EUS revealed that a heterogeneous tumor was located in the submucosal layer. The adventitia of esophagus and posterior of the trachea were infiltrated by tumor. Also, the paraesophageal lymph nodes greater than 10 mm in diameter appeared to be malignant. Biopsy specimens were obtained from the bulgy lesions. The cytopathologic analysis revealed gastric-like mucosa and poorly differentiated adenocarcinoma (Figure 2b). Thoracal computed tomography (CT) revealed a mass infiltrating into the wall of the anterior esophagus and several paraesophageal lymph nodes greater than 10 mm in diameter (T4, N1, M0). The condition was deemed to be inoperable. Cisplatin and radiotheraphy were suggested to the patient as treatment options by medical oncology. His nutrition situation was evaluated. A self-expanding metallic esophageal stent was attached to assist oral nutrition and then the patient was discharged. Unfortunately, he was lost to further follow-up.

## Discussion

In the literature there have been five reports of a hyperplastic polyp in the cervical esophagus [3-7]. In one case there was a large polypoidal mass causing dysphagia [3]. HGM in the esophagus has the potential for transformation into adenoma or adenosquamous carcinoma. But, to our knowledge, there have been no reports of malignant transformation of a polyp arising in HGM within the proximal esophagus. However, in the long-term study conducted by Uemura *et al*., 2.2% of gastric hyperplastic polyps eventually transformed into gastric cancer. Thus, the lesion in our patient was considered to carry a small risk of malignant transformation.

Adenocarcinomas of the cervical esophagus are rare. Faintuch *et al*. [8] reported a frequency of 1–2% for adenocarcinomas of the cervical esophagus. Adenocarcinomas of the esophagus can possibly arise from mucosal glands (cardiac glands), submucosal glands, heterotopic gastric mucosa and Barrett's esophagus. In contrast to Barrett's esophagus, HGM should not be regarded as a precancerous lesion. Immunohistochemical studies have demonstrated that inlet patches possess a distinctive embryonic gastric mucosa profile, while Barrett esophagus is considered an acquired condition that originates from immature gastrointestinal stem cells [9].

There are several reports of dysplasia or adenocarcinoma in heterotopic gastric mucosa within the esophagus. 24 cases were reported in the literature [1]. The patients with advanced carcinoma in the cervical esophagus may often require a pharyngo-laryngoesophagectomy depending on its location and clinical stage. In our patient, the carcinoma was not limited to the mucosal layer (T4N1M0) and was inoperable. Our case was HGM V according to the clinicopathological classification of von Rahden *et al*. [1]. The placement of a covered self-expanding metallic stent was achieved for the palliation of the patient's dysphagia and to assist oral nutrition. Esophageal stents have been used for several decades as part of palliative treatment of esophageal cancers. This procedure significantly relieves dysphagia and improves quality of life, which is the main therapeutic aim in inoperable cases.

## Conclusion

Patients with hyperplastic polyps in the esophagus need to be observed regularly, and we suggest that lesions that cause discomfort or increase in size, as in the case we have outlined, should be excised. Also biopsies should be taken from all identified cases of HGM. Surveillance with repeated biopsies is indicated only when intestinal metaplasia or dysplasia is seen. Endoscopic diagnosis of HGM is difficult and in daily clinical practice HGM is overlooked by many endoscopists. HGM is more commonly seen during withdrawal of the gastroscope. Therefore gastroenterologists should be aware of the possibility of HGM just distal to the upper esophageal sphincter.

## Competing interests

The author(s) declare that they have no competing interests.

## Authors' contributions

HA and ME have been involved in literature search, writing, conception, upper endoscopic contribution; MC made endoultrasonographic contribution and supported conception; SU and SD made final approval; AP and AD made pathological contribution.
